# Incentive and constraint regulations of rating inflation in collusion over the separation of economic cycles - Markov rating shopping dual reputation model

**DOI:** 10.1371/journal.pone.0205415

**Published:** 2018-10-17

**Authors:** Xiangyun Zhou, Yixiang Tian, Ping Zhang, Xiurong Chen

**Affiliations:** 1 School of Management and Economics, University of Electronic Science and Technology of China, Chengdu, China; 2 Purdue Technology Company Ltd of Shenzhen, Shenzhen, China; 3 School of Commerce and Economics, Zhengzhou University of Aeronautics, Zhengzhou, China; Shandong University of Science and Technology, CHINA

## Abstract

Economic cycles may lead to changes in corporate bond credit ratings. This paper utilizes the Markov model to describe transition probability matrixes of economic states for the separation of economic cycles. We develop a new model, which we term the Markov rating shopping dual reputation model, incorporating two reputation effects. This model is well suited to analyze the conditions of the dual rating incentive regulation and the constraint regulation for preventing rating inflation in collusion among credit rating agencies. Then, we apply the Markov regime switching-vector auto-regression (MS-VAR) to estimate the transition probability matrixes of America, England, Japan and China. Based on the numerical analysis and the simulations, the results show that a dual rating regulation can prevent the collusion of inflated ratings, as well as increased rating fees with the separation of economic cycles; additionally, when separating the economic cycles, a constraint regulation is more effective at reducing the risk of rating inflation in collusion and regulatory cost.

## Introduction

After the 2008 sub-prime crisis and the 2009 European debt crisis, credit rating agencies (CRAs) have been accused of assigning intentionally inflated ratings, i.e., giving good ratings to bad issuers [1]. For example, CRAs provided the highest rating AAA to the American International Group (AIG), but excessive speculation in the financial products caused the failure of AIG’s parent company in 2008. Based on the rating shopping mechanism [2]-[3], some researchers [4–7] show that rating shopping has created potential incentives to the assignation of rating inflation in order for CRAs’ to acquire more profit. In competitive rating industries, to minimize the conflicts of CRAs’ interests, CRAs may collude to assign inflated ratings and increase rating fees [8]-[9]. However, credit ratings can also be influenced by economic cycles. Regulators cannot detect whether rating inflation are caused by collusion or the economic cycles. As a result, it is unclear whether the current regulations induce CRAs to assign accurate ratings. In this article, we discuss the availability of regulations for rating inflation in collusion over the separation of economic cycles.

Some studies suggest that corporate bond ratings are influenced by the economic cycles. Auh suggests that rating inflation occurs more easily in booms [10]. Bar-Isaac and Shapiro J point to lower rating accuracy in boom states [11]. Nickell P et al use corporate bond ratings by Moody’s and find low-rated bonds are less prone to down-grades in business cycle peaks [12]. Bolton et al show that the accuracy of credit ratings may change in different economic environments [13]. We also provide empirical evidence that CRAs use the economic cycles in their bond ratings. Ryan P A et al utilize Moody’s upgrades and downgrades for corporate bonds from 1990 to 2010 [14]. Due to limited resource, we organize their data and show the relationship between bond ratings issued by Moody’s and American economic status.

**Fig 1:**
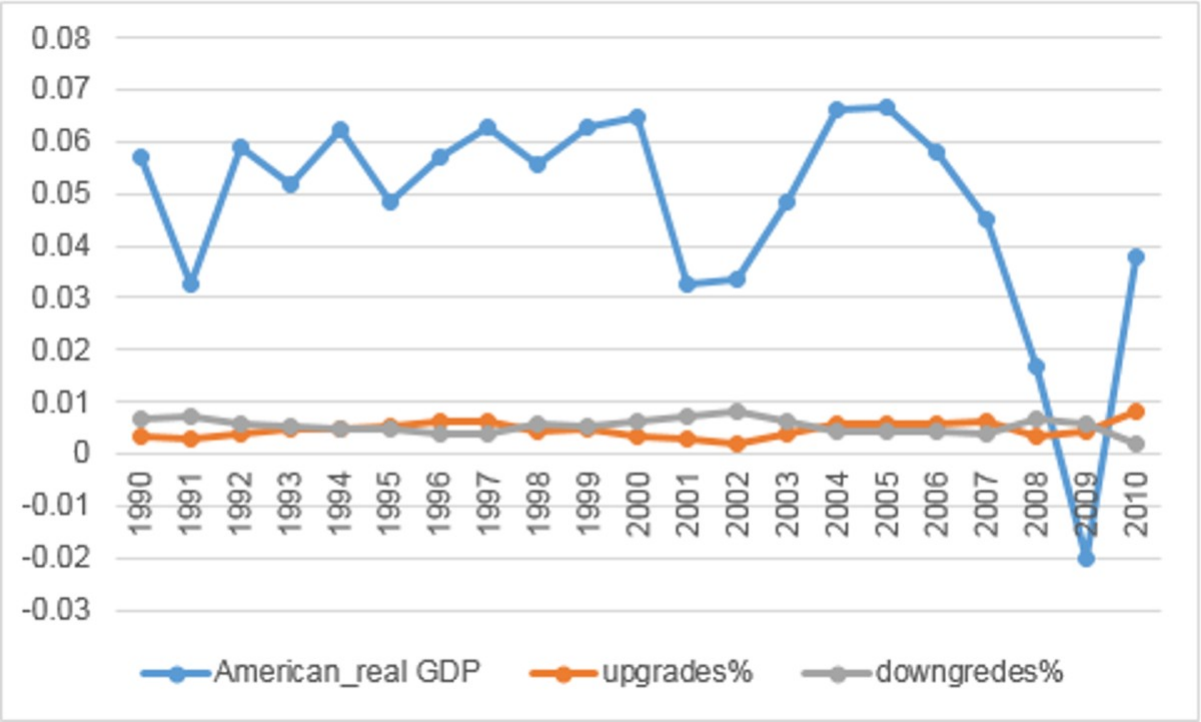
Moody’s rating changes and the American real GDP growth rates.

According to Fig 1, the general trend of rating upgrades is similar to that of the American real GDP growth rates, while the general trend of rating downgrades is opposite to that of the growth rates. This demonstrates that rating upgrades issued by Moody’s occur more easily in booms while rating downgrades occur in recessions. Due to the impact of economic cycles, it is hard for the regulators to design a series of regulatory policies, which can prevent rating inflation in collusion. In our model, we attempt to separate the changes in credit ratings from the effect of economic cycles.

Some researchers have showed the relationship between reputation effects and rating accuracy. Manso and Chen study two features of CRAs, namely, rating supervision and reliance on rating information for investors. CRAs may improve their reputation based on the two characteristics [15]. Frenkel reveals that CRAs have a “dual reputation effect”: institutional reputation and public reputation [16]. Regulators can utilize the regulatory penalty rate to induce CRAs to issue correct ratings [17]. Meanwhile, if CRAs offer inaccurate ratings, they will lose many buyers as a result of decreasing investment values [18]. Investors will “vote with their feet” and will punish the dishonest CRAs by reducing their market share [19]. We consider the dual reputation effect in our model.

There exist many empirical evidence [20–22] to show the relationship between ratings quality and regulations. A few of researchers focus on reasonable schemes. Opp et al theoretically analyze the repercussions on credit ratings implied by the Dodd-Frank Act [23]. However, Opp et al do not answer a broader question of optimal regulation design. Stolper considers approval schemes which can induce CRAs to offer correct ratings. We refer to these approval schemes and design a constraint scheme of collusion [8].

The regulators issued a dual rating requirement to solve the problem of rating inflation in many countries, such as America, England, Japan and China. In 1936, the Office of the Comptroller of the Currency (OCC) and the Federal Reserve issued the valuation principle that regulatory banks could not hold bonds which were not rated BBB or above by two CRAs [24]-[25]. In 1975, The Securities Exchange Act provided the net capital rules that ratings (BBB or above) from at least two CRAs were used as the basis of calculating the loss rates of risk assets of commercial bills and certificate of deposit held by broker leaders. In July 2010, Dodd-Frank act allowed investors and corporates who used ratings estimated the accuracy of ratings and compared with rating information issued by different CRAs. In 1993, the European Union (EU) required the dual rating system in perspective of investors and provided that debt instruments held by investment companies and trust companies were required to obtain ratings from at least two CRAs [24]. Although Brexit happened in 2016, the EU published the draft withdrawal agreement in February 2018. This draft required that England should comply with laws and rules of the EU during transition periods. Japanese regulatory authorities established a dual rating requirement in 1987. Japanese corporate bonds usually have two ratings: one rating can be provided by S&P or Moody’s, and the other should be issued by one of two domestic CRAs [26]. According to the 2013 self-discipline guidelines for non-financial corporate bond credit ratings, a dual rating requirement is positively encouraged when conducting business in China [27]. In 2013, the People’s Bank of China (PBoC) issued self-discipline guidelines for debt financing instruments of non-financial enterprises. According to the sixth provision of the guidelines, the dual ratings institution is firstly encouraged to practice in the rating market. Thus, we first discuss the available conditions of the dual rating regulation for preventing rating inflation in collusion to the four countries. We compare with the regulatory effect of the dual rating regulation and the constraint scheme.

This study develops a new model, the Markov rating shopping dual reputation model (Markov——RSDR) based on [8]. By contrast, we replace an economy shock with economic cycles in the model. To separate economic cycles, we utilize the Markov model to describe a transition probability matrix of economic states and incorporate this matrix into the model of [8]. We consider the dual reputation effect in the model. Investor’s and regulator’s penalty rates are described as public reputation and institutional reputation, respectively. We also add regulatory punishment of inflated ratings to CRAs’ revenues in accordance with realities. Meanwhile, we provide the available conditions of the dual rating regulation and the constraint mechanism; we estimate the transition probability matrixes of economic cycles using the MS-VAR model as one of variables in the numerical analysis and compare with the regulatory effect of the two regulations.

The rest of the paper is organized as follow. Section 2 introduces the model. Section 3 provides the available conditions of the dual rating incentives and the constraint regulations to prevent rating inflation in collusion. Section 4 shows the numerical analysis and the simulations. Section 5 makes conclusions and research prospects. The mathematical proofs, an example of the numerical analysis, the simulations of England, Japan and China are shown in the Appendix.

## The model

The assumptions of the model are similar to those of [8] and [13]. We consider a model with several CRAs, many issuers, a number of investors and a regulator. All of the participants are risk neutral. CRAs can observe the types of corporate bonds, high-quality bonds or low-quality bonds, but the regulator and investors cannot observe the types of bonds. Corporations issue bonds for a series of financing from investors. The process of rating is divided into four stages.

The first stage is one where the regulator approves some CRAs with access to the market and issue the dual rating regulation. We assume that three or more CRAs are in the market. There exist two types of corporate bonds, A and B. The real proportion of type-A bonds is *m*, where m∈(0,1). In period 0, $z_{i}^{t}$ denotes whether the regulator approves CRA *i* for the first time in period *t*. $z_{i}^{t}=1$, if the regulator approves CRA *i* in the rating market; otherwise, $z_{i}^{t}=0$. In this stage, the regulator has approval costs of *c_A_* for each CRA. $w_{i}^{t}$ represents whether CRA *i* qualifies for the provision of rating information. If CRA *i* can issue ratings, $w_{i}^{t}=1$. Otherwise, $w_{i}^{t}=0$. Every CRA has the regulatory cost $c_{M}$ in period *t*.

In the second stage, some CRAs decide on a rating fee and offer a rating to issuers. We suppose that CRA *i* chooses $f_{i}^{t}$ as its rating fee. The rating threshold of type-A corporate bonds is $\alpha_{i}^{t}$, namely, the number of rated type-A corporate bonds by CRAs. Accordingly, when $\alpha_{i}^{t}=m$, CRA *i* issues accurate ratings, then $\alpha_{i}^{t}>m$ and CRA *i* offers inflated ratings; however, when $\alpha_{i}^{t}<m$, CRA *i* provides deflated ratings. The ratings do not only depend on rating thresholds, it is also influenced by the economic cycles. Therefore, our model is introduced as a transition probability matrix of economic states *P* for separating economic cycles.

In the third stage, Issuers choose to buy two ratings from CRAs. An issuer’s utility of an A rating is *φ*, where *φ*>0. An issuer’s utility of a B rating is 0. The utility function of issuers is:

$$U_{is}=\{\begin{array}{c} \varphi -f_{i}^{t},\;\;\;\text{CRA}\;i\;\text{gives}\;A\;\text{rating}\\ -f_{i}^{t},\;\;\;\text{CRA}\;i\;\text{gives}\;B\;\text{rating}\\ 0,\;\;\;\text{It does not demand ratings} \end{array}$$ (1)

$D_{i}^{t}$ is the demand function of the credit ratings. When the CRA *i* is monopolistic, $D_{i}^{t}=\{\begin{array}{c} \alpha_{i}^{t},f_{i}^{t}<\varphi \\ 0,f_{i}^{t}>\varphi \end{array}$. We suppose other CRAs have the same rating fees $f_{-i}^{t}$ and an identical rating threshold $\alpha_{-i}^{t}$. In the competition of the CRAs, the demand function of CRA *i* is:

$$D_{i}^{t}=\{\begin{array}{c} \alpha_{i}^{t},f_{i}^{t}<f_{-i}^{t},f_{i}^{t}<\varphi \\ \frac{1}{n}\alpha_{i}^{t},f_{i}^{t}=f_{-i}^{t}\leq \varphi ,\alpha_{i}^{t}<\alpha_{-i}^{t}\\ \alpha_{i}^{t}-\frac{n-1}{n}\alpha_{-i}^{t},f_{i}^{t}=f_{-i}^{t}\leq \varphi ,\alpha_{i}^{t}>\alpha_{-i}^{t}\\ 0,\text{otherwise} \end{array}$$ (2)

In the fourth stage, several CRAs may provide inflated ratings in collusion and the regulator finds the collusion of rating inflation. To reduce the conflict among CRAs, they have an incentive for rating inflation and increased rating fees in collusion. In reality, CRAs should be punished for offering inaccurate ratings. We suppose there exist *n* CRAs to issue ratings in each year. The probability of collusion among these CRAs is *k*, and they should assume regulatory punishment $f_{i}^{t}\rho_{RE}$. Based on the dual reputation effect, Let *ρ*_*RA*_ and *ρ*_*RE*_ denote the investors’ penalty rate and the regulator’s penalty rate, respectively, where $\rho_{RA}\in [0,1]$ and $\rho_{RE}\in [0,1]$. With the separation of economic cycles, the regulator can quickly detect rating inflation in collusion by observing the default rates of corporate bonds. In fact, the default rates of type-A bonds *x^A^* are very low, while that of type-B bonds *x*^*B*^ are higher. If the default rates of type-A bonds rated by several CRAs are high, the regulator will give the regulatory punishment to these CRAs. In order to facilitate the analysis, we assume a rating inflation collusive phase and a punishment phase happen in period 1, which is different from [8].

From Eqs (3) and (4), the revenue function of CRA *i* and the cost function of the regulator are constructed by [8], where *σ*_*RA*_ and *σ*_*RE*_ represent the discount factors of CRAs and the regulator.

$${\bar{R}}_{RA,i}=(1-\sigma_{RA})\cdot \sum_{t=1}^{\infty }\sigma_{RA}^{t-1}\cdot D_{i}^{t}\cdot f_{i}^{t}$$ (3)

$${\bar{c}}_{RE}=(1-\sigma_{RE})\cdot \sum_{t=1}^{\infty }\sigma_{RE}^{t-1}\cdot [c_{A}\cdot \sum_{i}z_{i}^{t}+c_{M}\cdot \sum_{i}w_{i}^{t}]$$ (4)

In this model, Stolper considers without a common shock (e.g. an economy-wide shock) in the revenue function of CRAs and the cost function of the regulator. According to [1], we replace the common shock with economic cycles in the article. Actually, economic cycles have an effect on corporate bond ratings, which lead to the changes of the demand for bond ratings and the regulatory cost. Therefore, a common shock (economic cycles) should be considered in the model. Meanwhile, Stolper does not consider the regulatory punishment and the dual reputation effect.

In our model, we introduce a Markov transition probability matrix of economic states for the separation of economic cycles; we add the regulatory punishment to CRA *i*’s revenue and replace the discount factors with the dual reputation effect. The Markov rating shopping dual reputation model is:

The new revenue function of CRA *i*:

$$R_{\text{RA,i}}=(1-\rho_{\text{RA}})\cdot \sum_{t=1}^{\infty }\rho_{RA}^{t-1}\cdot D_{i}^{t}\cdot f_{i}^{t}\cdot P_{ij}^{t}-\sum_{t=1}^{\infty }k\cdot f_{i}^{t}\cdot \rho_{RE}$$ (5)

The new regulatory cost is:

$$c_{RE}=(1-\rho_{\text{RE}})\cdot \sum_{t=1}^{\infty }(c_{A}\cdot \sum_{i}^{n}z_{i}^{t}+c_{M}\cdot \sum_{i}^{n}w_{i}^{t})\cdot \rho_{RE}^{t-1}\cdot P_{ij}^{t}$$ (6)

The economic cycle *s* is usually divided into four periods: recession, depression, recovery and boom. In Eqs (5) and (6), the transition probability matrix of economic states is $P_{ij}=\left(\begin{array}{cccc} p_{11} & p_{12} & p_{13} & p_{14}\\ p_{21} & p_{22} & p_{23} & p_{24}\\ p_{31} & p_{32} & p_{33} & p_{34}\\ p_{41} & p_{42} & p_{43} & p_{44} \end{array}\right)$, where $p_{11}=p(s_{t+1}=1|s_{t}=1)$, $p_{12}=p(s_{t+1}=2|s_{t}=1)$, ……, $p_{44}=p(s_{t+1}=4|s_{t}=4)$.

Ross demonstrates that when *t* is close to infinity for all states *i*, $P_{ij}^{t}$ converges to a certain value [28]. In other words, there exists a limiting probability that we denote as *π*_*j*_, when an economic cycle transfers state *i* from state *j*.

We simplify Eqs (5) and (6):

$$R_{RA,i}=\pi_{j}\cdot \sum_{t=1}^{\infty }D_{i}^{t}\cdot f_{i}^{t}-\sum_{t=1}^{\infty }k\cdot f_{i}^{t}\cdot \rho_{RE}$$ (7)

$$c_{RE}=\pi_{j}\cdot \sum_{t=1}^{\infty }\left[c_{A}\cdot \sum_{i}^{n}z_{i}^{t}+c_{M}\cdot \sum_{i}^{n}w_{i}^{t}\right]$$ (8)

According to Eqs (7) and (8), the equilibrium results are the highest value of CRA *i*’s revenue function and the lowest value of regulatory cost.

Proof 1 is in S1 Appendix.

### Regulatory strategies

From the above analysis, CRAs probably collude to offer inflated ratings and higher rating fees. Based on the dual rating requirements, we discuss the availability of a dual rating regulation and a constraint regulation for preventing rating inflation in collusion. Additionally, we compare the regulatory effect over separation of economic cycles with the regulatory effect regardless of economic cycles.

#### The dual rating regulation for rating inflation in collusion

**Proposition 1.** There exists an investors’ penalty rate $\rho_{RA}\in (1-\frac{2\rho_{RE}}{\alpha_{i}-m\cdot \pi_{j}},1]$. In this situation, the regulator can utilize the dual rating regulation over the separation of economic cycles. However, without considering economic cycles, this regulation is ineffective at rating inflation.

In period 0, according to the dual rating regulation, the regulator requires issuers to buy two ratings from CRAs. We suppose CRA *i* and CAR *j* are selected by issuers at the same time. When the two CRAs offer accurate rating information without punishment, the rating thresholds and rating fees are *m* and *f*_*i*_ (*f*_*i*_<*φ*). Under the circumstances, the demand function of each CRA is $\frac{m}{2}$. In the separation of economic cycles, the expected revenue of each CRA is:

$$R_{1}=(1-\rho_{RA})\cdot \sum_{t=1}^{\infty }\rho_{RA}^{t-1}\cdot P_{ij}^{t-1}\cdot \frac{m}{2}\cdot f_{i}$$ (9)

When the rating threshold is *α*_*i*_∈(m,1], the CRAs collude to provide inflated ratings, but the rating fees are still *f*_*i*_. As the rating demands increase, the default probabilities of type-A corporate bonds are higher. We suppose the demand function of each CRA is $\frac{\alpha_{i}}{2}$ in period 1. Over the separation of economic cycles, the regulator is capable of distinguishing rating inflation in collusion. Then, the two CRAs will be punished by the regulator. Next, CRAs may issue accurate ratings and their demands are $\frac{m}{2}$. In this situation, the expected revenue of each CRA is:

$$R_{2}=(1-\rho_{RA})\cdot \frac{\alpha_{i}}{2}\cdot f_{i}+(1-\rho_{RA})\cdot \sum_{t=2}^{\infty }\rho_{RA}^{t-1}\cdot \frac{m}{2}\cdot f_{i}\cdot P_{ij}^{t-1}-f_{i}\cdot \rho_{RE}$$ (10)

To induce CRAs to offer accurate ratings, we let *R*_1_>*R*_2_ and we have:

$$1-\frac{2\rho_{RE}}{\alpha_{i}-m\cdot \pi_{j}}<\rho_{RA}\leq 1$$ (11)

Without the separation of economic cycles, the expected revenue of a CRA, which issues accurate ratings, is:

$$R_{1}^{'}=(1-\rho_{RA})\cdot \sum_{t=1}^{\infty }\rho_{RA}^{t-1}\cdot \frac{m}{2}\cdot f_{i}$$ (12)

In this case, there are difficulties for the regulator in recognizing the collusion of rating inflation. Thus, when the default rates are higher, CRAs may not be punished. CRAs can still offer inflated ratings. The expected revenue of a CRA in the collusion of rating inflation is:

$$R_{2}^{'}=(1-\rho_{RA})\cdot \sum_{t=1}^{\infty }\rho_{RA}^{t-1}\cdot \frac{\alpha_{i}}{2}\cdot f_{i}$$ (13)

According to Eqs (12) and (13), $R_{1}^{'}<R_{2}^{'}$ always holds. Therefore, a dual rating regulation is ineffective with respect to rating inflation without considering economic cycles.

For proof 2, please see S1 Appendix.

#### The dual rating regulation for rating inflation and higher rating fees in collusion

**Proposition 2.** With the separation of economic cycles, when the investors’ penalty rate $\rho_{RA}\in [1-\frac{2\cdot f\cdot \rho_{RE}}{\alpha_{i}\cdot \varphi -m\cdot f\cdot \pi_{j}},1-\frac{f\cdot \rho_{RE}}{\frac{\alpha_{i}}{2}\cdot \varphi -\frac{m}{n}\cdot f})$, the dual rating regulation can be used to address rating inflation and higher rating fees in collusion. However, regardless of economic cycles, this regulation has no effectiveness on rating inflation.

We assume that CRA *i* and CRA *j* are chosen by an issuer. For more revenues, the two CRAs may collude to inflate rating threshold *α*_*i*_ and may increase rating fees to *φ* (*f*_*i*_<*φ*). We suppose CRA *i* deviates from collusion, and it will not be punished. CRA *i* has accurate rating threshold *m* and lower rating fees *f*_*i*_. In this situation, some of the issuers may choose to buy the accurate ratings of CRA *i* for lower fees and reputation effects. According to the demand function of CRA *i*, when *m<α*_*i*_ and *f<φ*, we get $D_{i}=\frac{m}{n}$. When the rest of the CRAs are punished for rating inflation in collusion, they will offer accurate ratings in the next period. Over the separation of economic cycles, we assume the demand function of each CRA is $\frac{m}{2}$in period 2. The expected revenue of CRA *i* is:

$$R_{3}=(1-\rho_{RA})\cdot [(\frac{m}{n}\cdot f_{i}+\sum_{t=2}^{\infty }\rho_{RA}^{t-1}\cdot P_{ij}^{t-1}\cdot \frac{m}{2}\cdot f_{i}]$$ (14)

The expected revenue of each CRA in rating inflation is:

$$R_{4}=(1-\rho_{RA})\cdot [\frac{\alpha_{i}}{2}\cdot \varphi +\sum_{t=2}^{\infty }\rho_{RA}^{t-1}\cdot \frac{m}{2}\cdot f_{i}\cdot P_{ij}^{t-1}]-f_{i}\cdot \rho_{RE}$$ (15)

From Eqs (14) and (15), for *R*_4_>*R*_3_, we have:

$$\rho_{RA}<1-\frac{f\cdot \rho_{RE}}{\frac{\alpha_{i}}{2}\cdot \varphi -\frac{m}{n}\cdot f}$$ (16)

In this event, the CRAs’ revenues when colluding in rating inflation are higher. Thus, both CRAs will collude to offer overestimated ratings and higher rating fees.

In the comparison of Eqs (9) and (15), when *R*_1_≥*R*_4_, we get:

$$\rho_{RA}\geq 1-\frac{2\cdot f\cdot \rho_{RE}}{\alpha_{i}\cdot \varphi -m\cdot f\cdot \pi_{j}}$$ (17)

With the separation of economic cycles, the condition of a dual rating regulation for preventing rating inflation is:

$$1-\frac{2\cdot f\cdot \rho_{RE}}{\alpha_{i}\cdot \varphi -m\cdot f\cdot \pi_{j}}\leq \rho_{RA}<1-\frac{f\cdot \rho_{RE}}{\frac{\alpha_{i}}{2}\cdot \varphi -\frac{m}{n}\cdot f}$$ (18)

According to Eqs (9) and (14), when $\pi_{j}>\frac{2}{n}$ and *n*≥3, we can get *R*_1_>*R*_3_. This is a state of Nash Equilibrium in *ρ*_RA_∈[0,1]. In this case, if one CRA deviates from the collusion of rating inflation, all the CRAs in the market will issue accurate ratings for a maximum profit.

Without considering economic cycles, CRAs may not be punished. In this case, CRA *i* will collude at a certain time [8]. We assume that CRA *i* continues the collusion of rating inflation in period 2. The expected revenue of CRA *i*, which deviates from rating inflation is:

$$R_{3}^{'}=(1-\rho_{RA})\cdot [\frac{m}{n}\cdot f_{i}+\sum_{t=2}^{\infty }\rho_{RA}^{t-1}\cdot \frac{\alpha_{i}}{2}\cdot f_{i}]$$ (19)

The expected revenue of each CRA in rating inflation is:

$$R_{4}^{'}=(1-\rho_{RA})\cdot \sum_{i=1}^{\infty }\rho_{RA}^{t-1}\cdot \frac{\alpha_{i}}{2}\cdot \varphi$$ (20)

We can get $R_{4}^{'}>R_{3}^{'}$ from Eqs (19) and (20). For more revenue, all of the CRAs intend to collude, with no separation of economic cycles.

In the combination of Eqs (19) and (20), a dual rating regulation cannot be used to prevent the collusion of rating inflation without separating economic cycles.

Proof 3 can be seen in S1 Appendix.

#### The constraint regulation for rating inflation in collusion

**Proposition 3.** In period 0, the regulator introduces the collusion constraint regulation. We suppose the default probabilities of type-A corporate bonds, which are issued by CRA *j*, are higher. There is no approval of CRA *j* in the rating industry. Then, CRA *j* can be replaced by another CRA. In the separation of economic cycles, this regulation can reduce rating inflation.

CRA *i* deviates from rating inflation, its rating threshold is *m*, and its rating fee is *f*_*i*_. Nevertheless, CRA *j* still provides an inflated rating and a higher rating fee to *φ* (*f*_*i*_<*φ*). When the default rates *x*_*j*_>*x*_*i*_, the regulator denies approval to CRA *j*, and another CRA will replace it. In this case, the expected revenue of CRA *i* is also:

$$R_{5}=(1-\rho_{RA})\cdot (\frac{m}{n}\cdot f_{i}+\sum_{t=2}^{\infty }\rho_{RA}^{t-1}\cdot \frac{m}{2}\cdot f_{i}\cdot P_{ij}^{t-1})$$ (21)

Both CRA *i* and CRA *j* have rating thresholds *α*_*i*_ and rating fees *φ*. When the default probabilities of the rated type-A corporate bonds are higher, the regulator denies approval to the two CRAs. With the separation of economic cycles, we suppose the transition probability matrix of economic states is *P*_*ij*_ in the first period. The expected revenue of each CRA is:

$$R_{6}=\frac{\alpha_{i}}{2}\cdot \varphi \cdot P_{ij}-f_{i}\cdot \rho_{RE}$$ (22)

According to Eqs (21) and (22), we obtain $R_{5}>R_{6}$. This result shows that the constraint regulation can be used to reduce rating inflation. CRA *i* likely deviates from rating inflation for more revenues.

Similarly, regardless of economic states, the expected revenue of CRA *i* with deviation from the collusion of rating inflation is:

$$R_{5}^{'}=(1-\rho_{RA})\cdot [\frac{m}{n}\cdot f_{i}+\sum_{t=2}^{\infty }\rho_{RA}^{t-1}\cdot \frac{\alpha_{i}}{2}\cdot f_{i}]$$ (23)

In this event, it is difficult for the regulator to observe the collusion of rating inflation. Therefore, all of the CRAs cannot be punished. The expected revenue of each CRA involved in rating inflation is also:

$$R_{6}^{'}=(1-\rho_{RA})\cdot \sum_{t=1}^{\infty }\rho_{RA}^{t-1}\cdot \frac{\alpha_{i}}{2}\cdot \varphi_{i}$$ (24)

From Eqs (23) and (24), we have $R_{6}^{'}>R_{5}^{'}$. This result demonstrates that this regulation has no effectiveness in preventing rating inflation without considering economic cycles.

Please see proof 4 in S1 Appendix.

**Proposition 4.** When $\rho_{RE}\in [0,(1+\frac{c_{M}}{c_{A}}\cdot \pi_{j})-\frac{n+1}{2}\cdot \pi_{j}\cdot (1+\frac{c_{M}}{c_{A}})]$ and $c_{A}>c_{M}$, the constraint regulation has a lower regulatory cost with the separation of economic cycles.

The regulatory costs are comprised of $c_{A}$ and $c_{M}$. In the consideration of economic cycles, if the constraint regulation is not introduced, the regulatory cost will be:

$$c_{1}=(1-\rho_{RE})\cdot [n\cdot c_{A}+\sum_{t=1}^{\infty }\rho_{RE}^{t-1}\cdot P_{ij}^{t}\cdot n\cdot c_{M}]$$ (25)

In the constraint regulation, if a CRA failures to offer accurate ratings, it will be replaced with another CRA. The regulator should approve and regulate new CRAs. The regulatory cost in the constraint regulation will be:

$$\begin{array}{l} c_{2}=(1-\rho_{RE})\cdot [c_{A}\cdot n+\rho_{RE}\cdot P_{ij}\cdot c_{A}\cdot 1+\rho_{RE}^{2}\cdot P_{ij}^{2}\cdot c_{A}\cdot 2+\ldots \ldots +\rho_{RE}^{t-1}\cdot P_{ij}^{t-1}\cdot c_{A}\cdot (n-1)\\ +c_{M}\cdot n+\rho_{RE}\cdot P_{ij}\cdot c_{M}\cdot 1+\rho_{RE}^{2}\cdot P_{ij}^{2}\cdot c_{M}\cdot 2+\ldots \ldots +\rho_{RE}^{t-1}\cdot P_{ij}^{t-1}\cdot c_{M}\cdot (n-1)] \end{array}$$ (26)

$$c_{2}=(1-\rho_{RE})\cdot [\sum_{t=1}^{\infty }\rho_{RE}^{t-1}\cdot P_{ij}^{t}\cdot c_{A}\cdot \frac{n\cdot (n+1)}{2}+\sum_{t=1}^{\infty }\rho_{RE}^{t-1}\cdot P_{ij}^{t}\cdot c_{M}\cdot \frac{n\cdot (n+1)}{2}]$$ (27)

Accordingly, Eqs (25) and (27), when $c_{1}\geq c_{2}$, we have:

$$0\leq \rho_{RE}\leq (1+\frac{c_{M}}{c_{A}}\cdot \pi_{j})-\frac{n+1}{2}\cdot \pi_{j}\cdot (1+\frac{c_{M}}{c_{A}}),\;c_{1}>c_{2}$$ (28)

Please see proof 5 in the S1 Appendix.

The dual rating regulation has been put into practice in some countries and there is little effect on the regulatory cost for this regulation. However, the constraint regulation is newly designed. It is essential to examine the influence of regulatory results and costs. In summary, we obtain the following conclusions:

(1) In the separation of economic cycles, when the investors’ penalty rate $\rho_{RA}\in (1-\frac{2\rho_{RE}}{\alpha_{i}-m\cdot \pi_{j}},1]$, the dual rating regulation can be utilized to prevent the collusion of rating inflation. Without considering the economic cycles, this regulation has no effectiveness on rating inflation in collusion.
(2) Considering the economic states, when the investors’ penalty rate $\rho_{RA}\in [1-\frac{2\cdot f\cdot \rho_{RE}}{\alpha \cdot \varphi -m\cdot f\cdot \pi_{j}},1-\frac{f\cdot \rho_{RE}}{\frac{\alpha }{2}\cdot \varphi -\frac{m}{n}\cdot f})$, the collusion of inflating ratings and higher rating fees can be controlled by the dual rating regulation. Regardless of economic cycles, this regulation has a worse effect on rating inflation and increased rating fees.
(3) Compared with no separation of economic states, the constraint regulation is better able to reduce the collusion of inflated ratings in the separation of economic cycle.
(4) The regulator’s penalty rate $\rho_{RE}\in [0,(1+\frac{c_{M}}{c_{A}}\cdot \pi_{j})-\frac{n+1}{2}\cdot \pi_{j}\cdot (1+\frac{c_{M}}{c_{A}})]$ and $c_{A}>c_{M}$, the regulator can introduce the constraint regulation to reduce the regulatory costs.

## Results and discussion

The regression parameters of the nonlinear MS-VAR model [29] are dependent upon unobservable regime variables. These regime variables act in concert with the process of Markov-switching (MS). The MS-VAR model is better able to capture time-variant states of business cycles, and it discriminates the multistage transition processes of economic cycles. In this paper, the transition probability matrixes of economic states for America, England, Japan and China are estimated by this model. Then, we consider that the estimation results are important variables in the numerical analysis.

### Data processing

Using data of GDP for the 1960–2016 period in America, England, Japan and China from Wind database, we convert to the real GDP growth rate based on the GDP in 1978 [30]. We get $y_{t}$:

$$y_{t}=\frac{\text{The current real GDP}-\text{The previous real GDP}}{\text{The previous real GDP}}\text{*}100\text{\%}$$ (29)

This indicator can well measure the states of four countries’ economies [31]. For the availability of the MS-VAR model, we first test the unit root of $y_{t}$.

According to Table 1, all of the $y_{t}$ variables are stationary sequence. The real GDP growth rates can be dependent variables.

**Table 1:** Unit root tests of four countries’ real GDP growth rates $y_{t}$.

	*t*-statistics	*p*-values
Real GDP growth rate of America	-5.2912	0.0003
Real GDP growth rate of England	-4.6984	0.0020
Real GDP growth rate of Japan	-6.0374	0.0000
Real GDP growth rate of China	-5.5789	0.0001

Table 1 reports *t*-statistics and *p*-values for unit root tests of the four countries’ real GDP growth rates.

### MS-VAR model

The MS-VAR model can be written as:

$$y_{t}=\sum_{i=1}^{p}\phi_{i}(s_{t})\cdot y_{t-p}+\mu (s_{t})+u_{t}$$ (30)

$$u_{t}\sim iidN\left(0,\sigma^{2}\left(s_{t}\right)\right)$$ (31)

When an economic cycle occurs from state *i* to state *j*, the Markov transition probability matrix is:

$$P_{ij}=\left[\begin{array}{ccc} p_{11} & \ldots & p_{N1}\\ \vdots & \vdots & \vdots \\ p_{1N} & \cdots & p_{NN} \end{array}\right]$$ (32)

$$p_{ij}=P(s_{1,t}=j|s_{1,t-1}=i)$$ (33)

The specification in Eq (30) assumes that $y_{t}=(y_{1,t},y_{2,t},\ldots ,y_{n,t})$ denotes the real gross domestic product (GDP) growth rates. Through the empirical results, we decide that regime states $s_{t}$ correspond to economic cycles. For example, we assume that $s_{1,t}=j$ is one of four economic states. $\phi_{i}(s_{t})$, *i* = 1, 2, ……, *p*, are 4*4 regime dependent coefficient matrices, which are relative to $s_{t}$. $\mu (s_{t})$ is a 4*1 vector of regime dependent constant terms. $u_{t}$ is a 4*1 vector of stochastic error terms with zero mean and state dependent covariance matrix $\sigma^{2}(s_{t})$. Each of the four economies follows separated but potentially related regimes. Hence, we assume that:

$$\sigma^{2}(s_{t})=\left[\begin{array}{cccc} \sigma^{2}(s_{1,t}) & \rho (s_{1,t},s_{2,t})\sigma_{1}(s_{1,t})\sigma_{2}(s_{2,t}) & \rho (s_{1,t},s_{3,t})\sigma_{1}(s_{1,t})\sigma_{3}(s_{3,t}) & \rho (s_{1,t},s_{4,t})\sigma_{1}(s_{1,t})\sigma_{4}(s_{4,t})\\ \rho (s_{1,t},s_{2,t})\sigma_{1}(s_{1,t})\sigma_{2}(s_{2,t}) & \sigma^{2}(s_{2,t}) & \rho (s_{2,t},s_{3,t})\sigma_{2}(s_{2,t})\sigma_{3}(s_{3,t}) & \rho (s_{2,t},s_{4,t})\sigma_{2}(s_{2,t})\sigma_{4}(s_{4,t})\\ \rho (s_{1,t},s_{3,t})\sigma_{1}(s_{1,t})\sigma_{3}(s_{3,t}) & \rho (s_{2,t},s_{3,t})\sigma_{2}(s_{2,t})\sigma_{3}(s_{3,t}) & \sigma^{2}(s_{3,t}) & \rho (s_{3,t},s_{4,t})\sigma_{3}(s_{3,t})\sigma_{4}(s_{4,t})\\ \rho (s_{1,t},s_{4,t})\sigma_{1}(s_{1,t})\sigma_{4}(s_{4,t}) & \rho (s_{2,t},s_{4,t})\sigma_{2}(s_{2,t})\sigma_{4}(s_{4,t}) & \rho (s_{3,t},s_{4,t})\sigma_{3}(s_{3,t})\sigma_{4}(s_{4,t}) & \sigma^{2}(s_{4,t}) \end{array}\right]$$ (34)

In Eq (34), we suppose that the variance of the state variables for four economies ($\sigma^{2}(s_{t})$) depends on the economies’ own states. The parameter $\rho (s_{1,t},s_{2,t})$measures the correlation between the two of the four economic states.

We use MATLAB software to estimate the parameters of the MS-VAR model. From Akaike information criterion (AIC), we select the optimal lagged rank of a four regime MS-VAR model based on the smallest AIC value. The lagged rank should be two (Please see the results in S1A Table).

From Table 2, *ϕ*_1_ and *ϕ*_2_ denote the coefficient values of $y_{t-1}$ and $y_{t-2}$in four economic states, respectively. *σ*^2^ denotes state dependent covariance matrixes in the MS-VAR model. *P*_*ij*_ is the estimation of the Markov transition probability matrix. We focus on estimations of the four transition probability matrixes. The probabilities of the four countries staying in regime 1 are the highest. The probabilities of Japan in regime 1 is 95.20%. The probabilities of America and China in regime 4 are 4.73% and 34.21%, respectively. Additionally, the probabilities of England keeping regime 3 and regime 4 are 64.72% and 47.64%, respectively. However, in regime 2, the probabilities of the four countries are the lowest.

**Table 2:** Estimations of the MS-VAR model for America, England, Japan and China.

		*S*_*t*_ = 1	*S*_*t*_ = 2	*S*_*t*_ = 3	*S*_*t*_ = 4
America	$\phi_{1}$	0.6890***	-3.3168***	-4.6617***	5.1700***
	$\phi_{2}$	0.2700***	-1.0803***	1.4091***	0.1540***
	$\sigma^{2}$	0.00054***	0.00372***	0.01273***	0.02443***
	*P*	$P_{ij}=\left(\begin{array}{cccc} 1 & 0.2447 & 0.4289 & 0.0121\\ 0 & 0.1774 & 0.1661 & 0.1194\\ 0 & 0.2349 & 0.3837 & 0.8212\\ 0 & 0.3430 & 0.0213 & 0.0473 \end{array}\right)$			
England	$\phi_{1}$	0.6718	0.0477	1.0529***	0.0313
	$\phi_{2}$	-0.0945	0.0920	-0.0832***	0.0406
	$\sigma^{2}$	0.00724	0.00841	0.00049	0.02146
	*P*	$P_{ij}=\left(\begin{array}{cccc} 0.9484 & 0.9894 & 0.0036 & 0.0604\\ 0.0018 & 0.0103 & 0.2424 & 0.0617\\ 0 & 0 & 0.6472 & 0.4015\\ 0.0491 & 0 & 0.1068 & 0.4764 \end{array}\right)$			
Japan	$\phi_{1}$	0.5055***	3.1206***	0.3381***	1.1628***
	$\phi_{2}$	0.1430***	-1.1060***	-0.5774***	0.7147***
	$\sigma^{2}$	0.01073***	0.03227***	0.06321***	0.11507***
	*P*	$P_{ij}=\left(\begin{array}{cccc} 0.9520 & 1 & 0.3270 & 0.9922\\ 0 & 0 & 0.6726 & 0\\ 0.0015 & 0 & 0 & 0.0078\\ 0.0457 & 0 & 0 & 0 \end{array}\right)$			
China	$\phi_{1}$	0.8480***	-0.6431	1.0310	-0.6230
	$\phi_{2}$	-0.1337	-0.1215	0.5172	0.8026
	$\sigma^{2}$	0.00881	0.03616	0.00053	0.0001
	*P*	$P_{ij}=\left(\begin{array}{cccc} 0.9474 & 0.9341 & 0.5277 & 0.3084\\ 0 & 0.0658 & 0.1646 & 0.3469\\ 0.0181 & 0 & 0.2127 & 0.0027\\ 0.0345 & 0 & 0.0950 & 0.3421 \end{array}\right)$			

Note: ***, **, * The coefficient is statistically at the 1%, 5% and 10% levels, respectively.

### Numerical analysis

The estimations of the economic state transition probabilities matrixes are necessary variables in the numerical analysis. The number of CRAs *n* is based on the realities of the four countries’ rating industries. For example, the “Big Three” credit rating agencies provide ratings in America and England. In Japan, there exist two international CRAs, S&P and Moody’s, as well as two domestic CRAs, rating and investment information (R&I) and the Japan credit rating agency (JCR). Currently, the Chinese credit rating industry is still developing, with eight major CRAs, Dagong, Chenxin_Moody, Lianhe_Fitch, Jincheng, Lianhe, Pengyuan, Brilliance and Chengxin [32]. However, due to limited resources, some variables in this model are really difficult to find real data. These variables are the regulatory cost *C*_*M*_, the approval cost *C*_*A*_, Rating fee *f*_*i*_, the real number of type-A corporate bonds *m*. Meanwhile, some variables are assumptions, the increased rating fee in collusion *φ*_*i*_ and the inflated rating threshold in collusion *α*. Therefore, we give these variables random values. A set of parameter values are summarized in Table 3.

**Table 3:** Initial variables for the four countries.

Variables	America	England	Japan	China
The regulatory cost *C*_*M*_	30	25	20	10
The approval cost *C*_*A*_	35	30	25	20
Rating fee *f*_*i*_	20	20	15	10
Rating fee in collusion *φ*_*i*_	25	25	20	15
The real number of type-A corporate bonds *m*	(0,1/2)	(0,1/4)	(0,1/3)	(0,1/3)
The inflated rating threshold *α*	(1/2,1)	(1/4,1)	(1/3,1)	(1/3,1)
The number of CRAs *n*	3	3	4	8

Table 3 represents seven initial variables for America, England, Japan and China. The number of CRAs are based on the realities of the four countries. Meanwhile, the four Markov transition probability matrixes are reported in Table 2. Other variables are allocated by random values. We take America as an example and show how sensitive the findings are to the variables. According to Table 3 and $\rho_{RE}\in [0,(1+\frac{c_{M}}{c_{A}}\cdot \pi_{j})-\frac{n+1}{2}\cdot \pi_{j}\cdot (1+\frac{c_{M}}{c_{A}})]$, we can obtain the different values of *ρ*_*RE*_. In case of America, we get the value of $\rho_{RE}=(1+\frac{c_{M}}{c_{A}}\cdot \pi_{j})-\frac{n+1}{2}\cdot \pi_{j}\cdot (1+\frac{c_{M}}{c_{A}})$is $\left(\begin{array}{cccc} -1.8571 & 0.2923 & -0.2254 & 0.9654\\ 1 & 0.4931 & 0.5254 & 0.6589\\ 1 & 0.3289 & -0.0963 & -1.3463\\ 1 & 0.0200 & 0.9391 & 0.8649 \end{array}\right)$. Obviously, there are many available values of *ρ*_*RE*_ and it is difficult to list all of the situations. To save space, we only choose three groups *ρ*_*RE*_ for the numerical analysis in S1B Table. However, the simulations are based on available random values and describe the changes of CRAs’ revenues and the regulators’ cost. Meanwhile, we change random values of the initial variables in a control group in S1C Table and represent the examination of four propositions in S1D Table.

We compare with the two groups, although the values of the initial variables are different, the changes of CRAs’ revenues and regulators’ cost are similar and we can get the same results in accordance with the four propositions. Therefore, we use the sensitivity analysis to illustrate that the random values of some initial variables have no effectiveness on the examination of the propositions.

### Simulations

The simulations are used to examine the four propositions. We use X, Y and Z axes to illustrate CRAs’ expected revenues, *ρ*_*ra*_ and *ρ*_*re*_. Curved surfaces are described as the trends of CRAs’ expected revenues and the regulator’s cost with the changes of *ρ*_*ra*_ and *ρ*_*re*_. All figures are showed in Supporting Information file.

**Fig 2:**
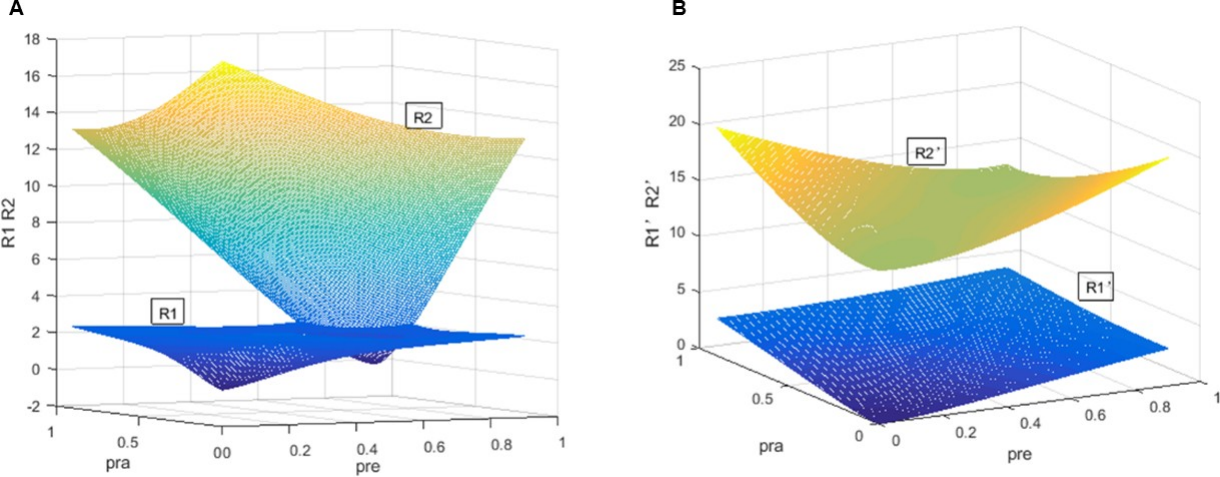
Comparison of *R*_*1*_, *R*_*2*_, *R*_*1*_*’*, *R*_*2*_*’* in America. (A) Comparison of *R*_*1*_, *R*_*2*_, *R*_*1*_*’*, *R*_*2*_*’* with the separation of economic cycles. (B) Comparison of *R*_*1*_, *R*_*2*_, *R*_*1*_*’*, *R*_*2*_*’* without considering the separation of economic cycles.

The illustration of dual rating regulation in America is shown in Fig 2. In Fig 2A, when the regulator chooses this regulation in the separation of economic cycles, we show the changes in the expected revenues of CRAs. With increasing regulators’ penalty rate *ρ*_*RE*_ and decreasing investors’ penalty rate *ρ*_*RA*_, a curved surface *R*_*1*_ increases while another curved surface *R*_*2*_ decreases. The two surfaces intersect in a curved line, which denotes *R*_*1*_ and *R*_*2*_ have the same values. After that, the expected revenue of accurate ratings *R*_*1*_ is higher than that of inflated ratings *R*_*2*_. To the opposite, regardless of economic cycles, the changes are shown in Fig 2B. A surface *R*_*2*_^*’*^ is still higher than another surface *R*_*1*_^*’*^. The two surfaces will never meet in three-dimensional space. For more revenues, when the regulator issues the dual rating regulation, CRAs can provide accurate ratings with the separation of economic cycles. Without considering economic cycles, the expected revenue of inflated ratings is still higher than that of accurate ratings. In this case, CRAs can collude to issue inflated ratings.

**Fig 3:**
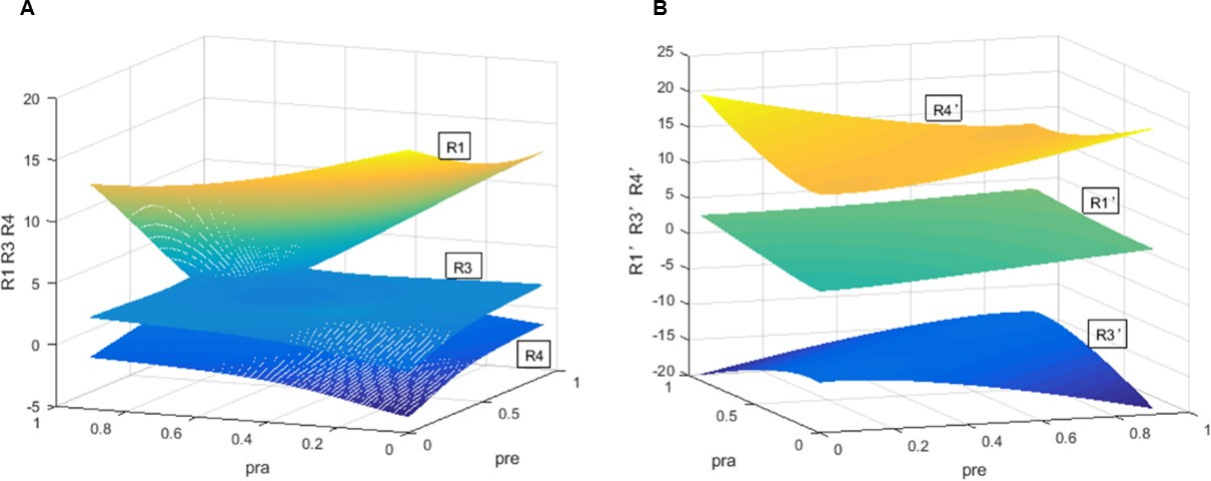
Comparison of *R*_*1*_, *R*_*3*_, *R*_*4*_, *R*_*1*_*’*, *R*_*3*_*’*, *R*_*4*_*’* in America. (A) Comparison of *R*_*1*_, *R*_*3*_, *R*_*4*_, *R*_*1*_*’*, *R*_*3*_*’*, *R*_*4*_*’* with the separation of economic cycles. (B) Comparison of *R*_*1*_, *R*_*3*_, *R*_*4*_, *R*_*1*_*’*, *R*_*3*_*’*, *R*_*4*_*’* without considering the separation of economic cycles.

According to Fig 3, under an available condition, the dual rating regulation can be used to prevent the collusion of rating inflation and higher rating fees. In Fig 3A, in the separation of economic cycles, with increasing regulators’ penalty rate *ρ*_*RE*_ and decreasing investors’ penalty rate *ρ*_*RA*_, the CRAs’ expected revenue when providing accurate ratings *R*_*1*_ and that of deviating from rating inflation *R*_*3*_ are increasing, but the CRAs’ expected revenue of rating inflation in collusion *R*_*4*_ is reducing. The three curved surfaces *R*_*1*_, *R*_*3*_ and *R*_*4*_ intersect in two curved lines, respectively. Then, *R*_*1*_ and *R*_*3*_ are all higher than *R*_*4*_. Meanwhile, *R*_*1*_ and *R*_*3*_ never meet in the space and *R*_*1*_ is still higher than *R*_*3*_. Without the separation of business cycles, in Fig 3B, we show that the three curved surfaces never meet. With the increasing *ρ*_*RE*_ and the decreasing *ρ*_*RA*_, *R*_*4*_*’* first decreases and then increases and *R*_*1*_*’* slowly increases, while *R*_*3*_*’* decreases. In this case, *R*_*1*_^*’*^ and *R*_*3*_^*’*^ are still lower than *R*_*4*_^*’*^ in *ρ*_*ra*_∈[0,1]. After issuing the dual regulation, CRAs can provide accurate ratings in the separation of economic cycles, while CRAs can issue inflated ratings in collusion without considering economic cycles. Meanwhile, when one CRA provides accurate ratings and other CRAs collude, the CRA has the lowest revenue. In this situation, no one will deviate from the collusion of rating inflation.

**Fig 4:**
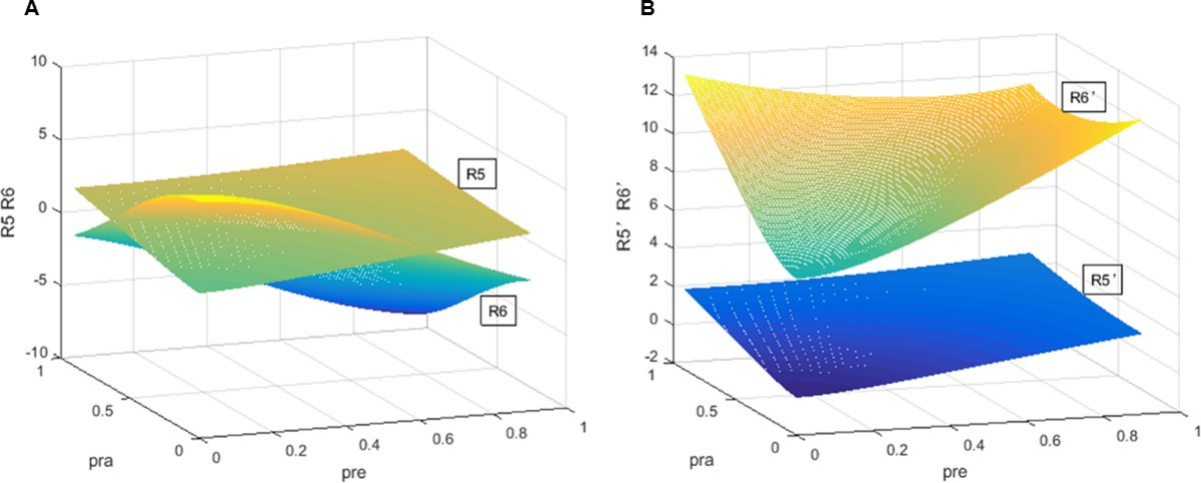
Comparison of *R*_*5*_, *R*_*6*_, *R*_*5*_*’*, *R*_*6*_*’* in America. (A) Comparison of *R*_*5*_, *R*_*6*_, *R*_*5*_*’*, *R*_*6*_*’* with the separation of economic cycles. (B) Comparison of *R*_*5*_, *R*_*6*_, *R*_*5*_*’*, *R*_*6*_*’* without considering the separation of economic cycles.

According to Fig 4, we describe the changes in the expected revenues of CRAs when the regulator utilizes the constraint regulation in America. Fig 4A shows the simulation results with the separation of economic cycles. With increasing regulators’ penalty rate *ρ*_*RE*_ and decreasing investors’ penalty rate *ρ*_*RA*_, the CRAs’ expected revenue of issuing accurate ratings *R*_*5*_ increases, while the CRAs’ expected revenue of rating inflation *R*_*6*_ decreases. The two curved surfaces *R*_*5*_ and *R*_*6*_ intersect in a curved line. Then, *R*_*5*_ is higher than *R*_*6*_. However, Fig 4B exhibits the situations without considering economic cycles. By contrast, with the increasing *ρ*_*RE*_ and the decreasing *ρ*_*RA*_, *R*_*5*_*’* and *R*_*6*_*’* increase. We discover that the CRAs’ expected revenue in rating inflation *R*_*6*_*’* is higher than that of accurate ratings *R*_*5*_*’* in *ρ*_*ra*_∈[0,1]. Additionally, the two curved surfaces never meet in the space. After introducing the constraint regulation, CRAs’ expected revenue of accurate ratings will be higher than that of inflated ratings in the separation of economic cycles. It demonstrates that the constraint regulation can reduce CRA to provide accurate ratings with considering economic cycles.

**Fig 5:**
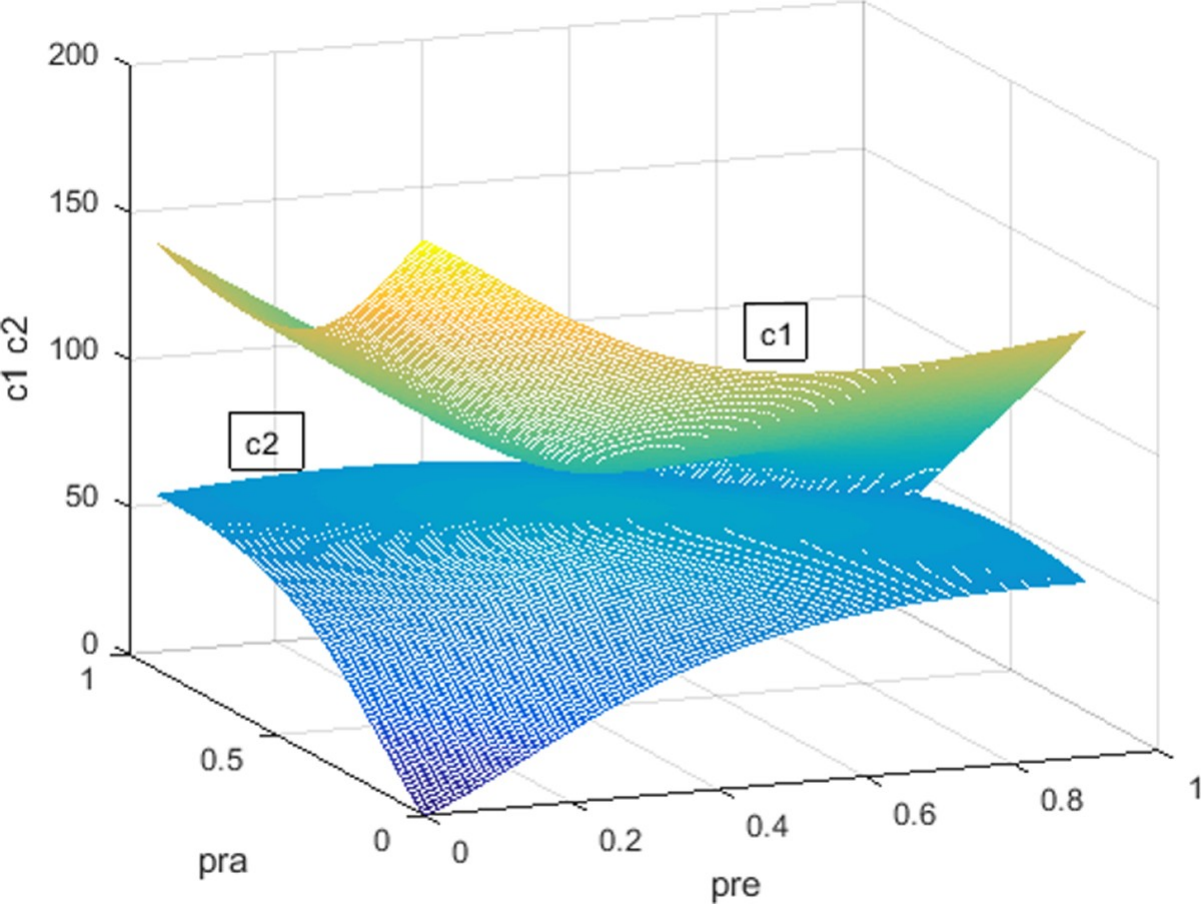
Comparison of *c*_*1*_, *c*_*2*_ in America.

According to Fig 5, with increasing regulator’s penalty rate *ρ*_*re*_ and decreasing investors’ penalty rate *ρ*_*RA*_, the cost of the regulator in the constraint regulation *c*_*2*_ first increases and then decreases. The regulator’s cost out of the constraint regulation *c*_*1*_ decreases. However, *c*_*2*_ is still lower than *c*_*1*_. It demonstrates that the constraint regulation can reduce the regulator’s cost. The same results are shown in England, Japan and China, respectively.

Overall, Fig 2 presents for the first proposition. Fig 3 is testing the second proposition. The third proposition is proven in Fig 4. Finally, Fig 5 shows the last proposition. The constraint regulation is first proposed and introduced in this thesis. In the last figure, we examine the impact of the constraint regulation on the regulatory cost. If this scheme has a negative effect on the regulatory cost, it will not be an available scheme for the regulators. Meanwhile, the dual rating regulation has been adopted in many countries. It is not necessary to show the regulatory cost of the dual rating regulation. The changes of CRAs’ revenues are similar in England, Japan and China. Please see the simulations of three countries in S1, S2 and S3 Figs. The simulations demonstrate the dual rating regulation has a regulatory effect within limits and the constraint regulation is better for reducing the collusion risk of rating inflation and the regulator’s cost with the separation of economic cycles. However, regardless of economic cycles, the dual rating incentive regulation and the constraint regulation have no effectiveness on the collusion of rating inflation.

## Conclusions and research prospect

This paper discusses the dual rating incentives and the constraint regulations for preventing the collusion of rating inflation in the separation of economic cycles. Based on [8], we develop a new model and provide the available conditions of the two regulations. In addition, the model, the numerical analysis and the simulations show the following:

1. We find that economic cycles have a great influence on bond ratings. Therefore, we introduce a transition probability matrix of economic states in the model. Meanwhile, according to the dual reputation effect on CRAs, the investor’s penalty rates and the regulator’s penalty rates are described as the dual reputation. Finally, we develop a new model, namely, the Markov rating shopping dual reputation model. This model is closer to realities.

2. We utilize the MS-VAR model to estimate four countries’ transition probability matrixes of economic states. These matrixes are important variables in the numerical analysis. Through the four countries’ simulations of the dual rating regulation and the constraint regulation, we can represent that the four propositions are universal.

3. Our results show that in the separation of economic cycles, the dual rating regulation can be used to prevent rating inflation in collusion within limits. Additionally, a constraint regulation should be more effective at reducing the collusion risk of rating inflation and regulatory cost. However, without considering economic cycles, the two regulations are ineffective.

In the present paper, we only discuss the available conditions of the dual rating incentives and the constraint regulations to prevent rating inflation in view of the long-term perspective. According to [1], we can discuss rating inflation from the short-term perspective. Additionally, we will consider other measures, such as banks’ internal ratings and will provide more empirical evidence in further research.

## Supporting information

S1 Data This is the data set for the research. (RAR)

S1 Appendix This is the supplement of the research. (DOCX)

S1 Table These are additional tables of the research. (DOCX)

S1 Fig These are simulations in England. Fig 1 is comparison of *R*_*1*_, *R*_*2*_, *R*_*1*_*’*, *R*_*2*_*’*. Fig 2 is comparison of *R*_*1*_, *R*_*3*_, *R*_*4*_, *R*_*1*_*’*, *R*_*3*_*’*, *R*_*4*_*’*. Fig 3 is comparison of *R*_*5*_, *R*_*6*_, *R*_*5*_*’*, *R*_*6*_*’*. Fig 4 is comparison of *c*_*1*_, *c*_*2*_.
(RAR)

S2 Fig These are simulations in Japan. Fig 1 is comparison of *R*_*1*_, *R*_*2*_, *R*_*1*_*’*, *R*_*2*_*’*. Fig 2 is comparison of *R*_*1*_, *R*_*3*_, *R*_*4*_, *R*_*1*_*’*, *R*_*3*_*’*, *R*_*4*_*’*. Fig 3 is comparison of *R*_*5*_, *R*_*6*_, *R*_*5*_*’*, *R*_*6*_*’*. Fig 4 is comparison of *c*_*1*_, *c*_*2*_.
(RAR)

S3 Fig These are simulations in China. Fig 1 is comparison of *R*_*1*_, *R*_*2*_, *R*_*1*_*’*, *R*_*2*_*’*. Fig 2 is comparison of *R*_*1*_, *R*_*3*_, *R*_*4*_, *R*_*1*_*’*, *R*_*3*_*’*, *R*_*4*_*’*. Fig 3 is comparison of *R*_*5*_, *R*_*6*_, *R*_*5*_*’*, *R*_*6*_*’*. Fig 4 is comparison of *c*_*1*_, *c*_*2*_.
(RAR)
