# Supplementary material for: Incentive and constraint regulations of rating inflation in collusion over the separation of economic cycles - Markov rating shopping dual reputation model
Source: PLoS One. 2018 Oct 17;13(10):e0205415. doi: 10.1371/journal.pone.0205415 (PMC6192634; doi:10.1371/journal.pone.0205415)
Supplement: S1 Appendix — (DOCX) [file pone.0205415.s002.docx]

**Theorem 1:** For an irreducible ergodic Markov chain $\lim_{t\to\infty}P_{ij}^{t}$ exists and is independent of *i*. Therefore, we let$\pi_{j}=\lim_{t\to\infty}P_{ij}^{t}$, then $\pi_{j}$ is the unique nonnegative solution:

$$\pi_{j}=\sum_{i=0}^{\infty} \pi_{i}\cdot P_{ij}$$

Where *j*≥0 and$\sum_{j=0}^{i} \pi_{j}=1$.

**Remark:** We suppose $\lim_{t\to\infty}P_{ij}^{t}$exists and is independent of the initial state *i*. Let us derive an expression for $P\left\{s_{t+1}=j\right\}$by conditioning on the state at time *t*. We can obtain:

$$P\left\{s_{t+1}=j\right\}=\sum_{i=0}^{\infty} P\left\{s_{t+1}=j\text{|}s_{t}=i\right\}\cdot P\left\{s_{t}=i\right\}$$

$$P\left\{s_{t+1}=j\right\}=\sum_{i=0}^{\infty} P_{ij}P\left\{s_{t}=i\right\}$$

Letting$t\to\infty$, we can bring the limit inside the summation, leads to$\pi_{j}=\sum_{i=0}^{\infty} \pi_{i}\cdot P_{ij}$.

**Proof 1:** Due to Theorem 1, and the following equations:

$$\sum_{t=1}^{\infty} \rho_{RA}^{t-1}=\lim_{t\to\infty} \frac{1-\rho_{RA}^{t}}{1-\rho_{RA}}=\frac{1}{1-\rho_{RA}}$$

$$\sum_{t=2}^{\infty} \rho_{RA}^{t-1}=\lim_{t\to\infty} \frac{\rho_{RA}-\rho_{RA}^{t}}{1-\rho_{RA}}=\frac{\rho_{RA}}{1-\rho_{RA}}$$

We can simplify the equations of CRAs’ revenues and the regulator’ cost, then we have:

$$R_{RA,i}=\pi_{j}\cdot\sum_{t=1}^{\infty} D_{i}^{t}\cdot f_{i}^{t}-\sum_{t=1}^{\infty} k\cdot f_{i}^{t}\cdot\rho_{RE}$$

**Proof 2 of proposition 1:**

$$R_{1}=(1-\rho_{RA})\cdot\sum_{t=1}^{\infty} \rho_{RA}^{t-1}\cdot P_{ij}^{t-1}\cdot\frac{m}{2}\cdot f_{i}=\pi_{j}\cdot\frac{m}{2}\cdot f$$

$$R_{2}=(1-\rho_{RA})\cdot\frac{\alpha_{i}}{2}\cdot f_{i}+(1-\rho_{RA})\cdot\sum_{t=2}^{\infty} \rho_{RA}^{t-1}\cdot\frac{m}{2}\cdot f_{i}\cdot P_{ij}^{t-1}-f_{i}\cdot\rho_{RE}$$

$$\begin{matrix} R_{2}=(1-\rho_{RA})\cdot\frac{\alpha_{i}}{2}\cdot f_{i}+\rho_{RA}\cdot\frac{m}{2}\cdot f\cdot\pi_{j}-f_{i}\cdot\rho_{RE} \\ \end{matrix}$$

Letting$R_{1}>R_{2}$, we have:

$$\rho_{RA}\cdot(\frac{\alpha_{i}}{2}-\frac{m}{2}\cdot\pi_{j})>\frac{\alpha_{i}}{2}-\frac{m}{2}\cdot\pi_{j}-\rho_{RE}$$

$$\rho_{RA}>1-\frac{2\rho_{RE}}{\alpha_{i}-m\cdot\pi_{j}}$$

In this assumption, the investors’ penalty rate$\rho_{RA}\leq1$. Therefore, we get$\rho_{RA}\in(1-\frac{2\rho_{RE}}{\alpha_{i}-m\cdot\pi_{j}},1]$.

$$R_{1}^{'}=(1-\rho_{RA})\cdot\sum_{t=1}^{\infty} \rho_{RA}^{t-1}\cdot\frac{m}{2}\cdot f_{i}=\frac{m}{2}\cdot f_{i}$$

$$R_{2}^{'}=(1-\rho_{RA})\cdot\sum_{t=1}^{\infty} \rho_{RA}^{t-1}\cdot\frac{\alpha_{i}}{2}\cdot f_{i}=\frac{\alpha_{i}}{2}\cdot f_{i}$$

$$R_{2}^{'}-R_{1}^{'}=(\frac{\alpha_{i}}{2}-\frac{m}{2})\cdot f_{i}$$

Because of$\alpha_{i}>m$, $R_{2}^{'}>R_{1}^{'}$is constant.

**Proof 3 of proposition 2:**

$$R_{3}=(1-\rho_{RA})\cdot[(\frac{m}{n}\cdot f_{i}+\sum_{t=2}^{\infty} \rho_{RA}^{t-1}\cdot P_{ij}^{t-1}\cdot\frac{m}{2}\cdot f_{i}]=(1-\rho_{RA})\cdot\frac{m}{n}\cdot f_{i}+\rho_{RA}\cdot\pi_{j}\cdot\frac{m}{2}\cdot f_{i}$$

For$R_{4}>R_{3}$, we have:

Because of$\frac{\alpha_{i}}{2}>\frac{m}{n}$and, we obtain:

If $R_{1}\geq R_{4}$, we can get:

Due to, it is easy to testify:

Finally, both CRAs will collude to inflate ratings and to increase rating fees if and only if .

Meanwhile, if $R_{1}>R_{3}$, we will have:

$$\frac{m}{2}\cdot f_{i}\cdot\pi_{j}>(1-\rho_{RA})\cdot\frac{m}{n}\cdot f_{i}+\rho_{RA}\cdot\frac{m}{2}\cdot f_{i}\cdot\pi_{j}$$

$$(1-\rho_{RA})\cdot\frac{m}{2}\cdot f_{i}\cdot\pi_{j}>(1-\rho_{RA})\cdot\frac{m}{n}\cdot f_{i}$$

Solve it and we get:

$$\pi_{j}>\frac{2}{n}$$

Therefore, all of the CRAs in the market will offer accurate ratings if and only if $\pi_{j}\in(\frac{2}{n},1]$, where$n\geq3$.

$$R_{3}^{'}=(1-\rho_{RA})\cdot[\frac{m}{n}\cdot f_{i}+\sum_{t=2}^{\infty} \rho_{RA}^{t-1}\cdot\frac{\alpha_{i}}{2}\cdot f_{i}]=(1-\rho_{RA})\cdot\frac{m}{n}\cdot f+\rho_{RA}\cdot\frac{\alpha_{i}}{2}\cdot f_{i}$$

We suppose, where$\varepsilon>0$; thus, we have:

$$R_{4}^{'}-R_{3}^{'}=(1-\rho_{RA})\cdot f_{i}\cdot(\frac{\alpha_{i}}{2}-\frac{m}{n})+\frac{\alpha_{i}}{2}\cdot\varepsilon$$

Obviously, $R_{4}^{'}$is always higher than$R_{3}^{'}$

**Proof 4 of proposition 3:**

$$R_{5}=(1-\rho_{RA})\cdot(\frac{m}{n}\cdot f_{i}+\sum_{t=2}^{\infty} \rho_{RA}^{t-1}\cdot\frac{m}{2}\cdot f_{i}\cdot P_{ij}^{t-1})=(1-\rho_{RA})\cdot\frac{m}{n}\cdot f+\rho_{RA}\cdot\frac{m}{2}\cdot f\cdot\pi_{j}$$

If and only if $\pi_{j}\in(\frac{2}{n},1]$, we have:

$$\frac{\partial\Delta}{\partial\rho_{RA}}=m\cdot f_{i}\cdot(\frac{\pi_{j}}{2}-\frac{1}{n})>0$$

$\Delta$is monotonically increasing in$\rho_{RA}\in[0,1]$. Therefore, when$\rho_{RA}=0$, $\Delta$can attach minimum value.

If$\rho_{RA}=0$, it will clearly show.Therefore, we get$R_{5}>R_{6}$.

Without consideration of economic cycles,

$$R_{5}^{'}=(1-\rho_{RA})\cdot[\frac{m}{n}\cdot f_{i}+\sum_{t=2}^{\infty} \rho_{RA}^{t-1}\cdot\frac{\alpha_{i}}{2}\cdot f_{i}]=(1-\rho_{RA})\cdot\frac{m}{n}\cdot f_{i}+\rho_{RA}\cdot\frac{\alpha_{i}}{2}\cdot f_{i}$$

It is noticeable that$R_{6}^{'}>R_{5}^{'}$.

**Proof 5 of proposition 4:**

$$c_{1}=(1-\rho_{RE})\cdot[n\cdot c_{A}+\sum_{t=1}^{\infty} P_{ij}^{t}\cdot n\cdot c_{M}\cdot\rho_{RE}^{t-1}]=(1-\rho_{RE})\cdot n\cdot c_{A}+\pi_{j}\cdot n\cdot c_{M}$$

$$c_{2}=(1-\rho_{RE})\cdot[\sum_{t=1}^{\infty} \rho_{RE}^{t-1}\cdot P_{ij}^{t}\cdot c_{A}\cdot\frac{n\cdot(n+1)}{2}+\sum_{t=1}^{\infty} \rho_{RE}^{t-1}\cdot P_{ij}^{t}\cdot c_{M}\cdot\frac{n\cdot(n+1)}{2}]=\pi_{j}\cdot\frac{n(n+1)}{2}\cdot(c_{A}+c_{M})$$

When$c_{1}\geq c_{2}$, we have:

$$c_{A}+c_{M}\cdot\pi_{j}-\frac{n+1}{2}\cdot\pi_{j}\cdot(c_{A}+c_{M})\geq c_{A}\cdot\rho_{RE}$$

$$0\leq\rho_{RE}\leq(1+\frac{c_{M}}{c_{A}}\cdot\pi_{j})-\frac{n+1}{2}\cdot\pi_{j}\cdot(1+\frac{c_{M}}{c_{A}})$$
