# Supplementary material for: Incentive and constraint regulations of rating inflation in collusion over the separation of economic cycles - Markov rating shopping dual reputation model
Source: PLoS One. 2018 Oct 17;13(10):e0205415. doi: 10.1371/journal.pone.0205415 (PMC6192634; doi:10.1371/journal.pone.0205415)
Supplement: S1 Table — (DOCX) [file pone.0205415.s003.docx]

**Table A AIC of lagged ranks**

|  | Lagged rank  *n*=2 | Lagged rank  *n*=3 | Lagged rank  *n*=4 |
| --- | --- | --- | --- |
| America_ AIC | -214.2513 | -209.7994 | -202.9696 |
| England_ AIC | -62.5738 | -47.9515 | -40.0268 |
| Japan_ AIC | -33.3121 | -26.0498 | -22.5602 |
| China_ AIC | -68.0520 | -62.4256 | -38.4807 |

**Table B Numerical analysis of three group *ρ_RE_* in America**

| *ρ_RE_* | 0.9654 | 0.5254 | 0.2923 |
| --- | --- | --- | --- |
| *ρ_RA_* | 0.0017 | 0.8683 | 1 |
| *R_1_* | (0 2.5143 1.4961 2.8555) | (0 2.5143 1.4961 2.8555) | (0 2.5143 1.4961 2.8555) |
| *R_2_* | (4.9915 0.4932 -4.0111 -8.5113) | (0.6587 0.0394 -3.6471 -5.2691) | (0 2.3943 1.2561 2.4955) |
| *R_1_’* | (0 1.5 3 4.5) | (0 1.5 3 4.5) | (0 1.5 3 4.5) |
| *R_2_’* | (4.9915 6.4915 7.9915 9.4915) | (0.6587 2.1587 3.6587 5.1587) | (0 1.5 3 4.5) |
| *ρ_RA_* | 0.3294 | 0.3353 | 0.6455 |
| *R_1_* | (0 2.5143 1.4961 2.8555) | (0 2.5143 1.4961 2.8555) | (0 2.5143 1.4961 2.8555) |
| *R_3_* | (-4.4707 -2.9719 -2.6367 -1.5182) | (-4.4313 -2.9236 -2.6003 -1.4798) | (-2.3633 -0.3859 -0.6886 0.5434) |
| *R_4_* | (3.3530 -0.8129 -6.1424 -10.6887) | (3.3235 2.1636 -0.1808 -1.7279) | (1.7725 2.3272 0.6017 0.4110) |
| *R_1_’* | (0 1.5 3 4.5) | (0 1.5 3 4.5) | (0 1.5 3 4.5) |
| *R_3_’* | (-4.4707 -3.3060 -2.1413 -0.9766) | (-4.4313 -3.2637 -2.0960 -0.9284) | (-0.8627 0.5726 2.0079 3.4432) |
| *R_4_’* | (3.3530 4.8530 6.3530 7.8530) | (3.3235 4.8235 6.3235 7.8235) | (0.6407 2.1470 3.6470 5.1470) |
| *ρ_RA_* | 0.1 | 0.5 | 0.85 |
| *R_5_* | (0 1.0676 1.8997 2.8904) | (0 1.3381 1.4987 2.4519) | (0 1.5748 1.1478 2.0681) |
| *R_6_* | (6.2500 3.4116 -3.8799 -8.1806) | (6.2500 6.4116 2.1201 0.8194) | (6.2500 7.8116 4.9201 5.0194) |
| *R_5_’* | (0 1 2 3) | (0 1 2 3) | (0 1 2 3) |
| *R_6_’* | (6.2500 8.1250 10 11.8750) | (6.2500 8.1250 10 11.8750) | (6.2500 8.1250 10 11.8750) |
| *c_1_* | (390 96.6030 167.2710 4.7190) | (139.8330 72.1260 88.4340 50.9220) | (164.3085 96.6015 112.9095 75.3975) |
| *c_2_* | (93.6330 25.9260 42.2340 4.7220) | (0 69.1860 64.7790 46.5660) | (0 69.1860 64.7790 46.5660) |

**Table C Values of initial variables in the model for America**

| Variables | American |
| --- | --- |
| The regulatory cost *C_M_* | 10 |
| The approval cost *C_A_* | 12 |
| Rating fee$f_{i}$ | 15 |
| Rating fee in collusion | 18 |
| The real number of type-A corporate bonds *m* | (0,1/3) |
| The inflated rating threshold in collusion *α* | (1/3,1) |
| The number of CRAs *n* | 3 |

According to Table C and$\rho_{RE}\in[0,(1+\frac{c_{M}}{c_{A}}\cdot\pi_{j})-\frac{n+1}{2}\cdot\pi_{j}\cdot(1+\frac{c_{M}}{c_{A}})]$, we get the value of $\rho_{RE}=(1+\frac{c_{M}}{c_{A}}\cdot\pi_{j})-\frac{n+1}{2}\cdot\pi_{j}\cdot(1+\frac{c_{M}}{c_{A}})$is$\left(\begin{matrix} -1.8333 & 0.2982 & -0.2152 & 0.9657 \\ 1 & 0.4974 & 0.5294 & 0.6617 \\ 1 & 0.3345 & -0.0872 & -1.3267 \\ 1 & 0.0282 & 0.9396 & 0.8660 \end{matrix}\right)$ and only choose the following three groups *ρ_RE_* in Table D.

**Table D Numerical analysis of other three group *ρ_RE_* in America**

| *ρ_RE_* | 0.9657 | 0.5294 | 0.3345 |
| --- | --- | --- | --- |
| *ρ_RA_* | 0.1156 | 0.8396 | 1 |
| *R_1_* | (0 1.2572 0.7481 1.4278) | (0 1.2572 0.7481 1.4278) | (0 1.2572 0.7481 1.4278) |
| *R_2_* | (2.2110 -0.9497 -4.3146 -7.5421) | (0.4010 -0.5770 -3.0379 -4.5006) | (0 -0.2428 -2.2520 -3.0722) |
| *R_1_’* | (0 0.75 1.5 2.25) | (0 0.75 1.5 2.25) | (0 0.75 1.5 2.25) |
| *R_2_’* | (2.2110 3.4916 4.7723 6.0529) | (0.4010 1.2472 2.0935 2.9397) | (0 0.75 1.5 2.25) |
| *ρ_RA_* | 0.3516 | 0.6758 | 0.9005 |
| *R_1_* | (0 1.2572 0.7481 1.4278) | (0 1.2572 0.7481 1.4278) | (0 1.2572 0.7481 1.4278) |
| *R_3_* | (-2.1613 -1.9138 -2.2874 -2.2429) | (-1.0807 -0.3283 -0.7697 -0.4076) | (-0.3317 0.7705 0.2823 0.8645) |
| *R_4_* | (1.6210 -1.5616 -5.3653 -8.7510) | (0.8105 -0.1522 -2.3086 -3.6616) | (0.2488 0.0151 -1.8090 -2.5626) |
| *R_1_’* | (0 0.75 1.5 2.25) | (0 0.75 1.5 2.25) | (0 0.75 1.5 2.25) |
| *R_3_’* | (-2.1613 -2.0922 -2.0230 -1.9538) | (-1.0807 -0.6711 -0.2615 0.1481) | (-0.3317 0.3139 0.9594 1.6049) |
| *R_4_’* | (1.6210 2.7600 3.8991 5.0381) | (0.8105 1.7550 2.6995 3.6441) | (0.2488 1.0585 1.8682 2.6779) |
| *ρ_RA_* | 0.1 | 0.5 | 0.85 |
| *R_5_* | (0 0.5338 0.9499 1.4452 ) | (0 0.6691 0.7494 1.2259) | (0 0.7874 0.5739 1.0341) |
| *R_6_* | (3 1.2244 -4.3842 -7.4160) | (3 3.4744 0.1158 -0.6660) | (3 4.2244 1.6158 1.5840) |
| *R_5_’* | (0 0.5 1 1.5) | (0 0.5 1 1.5) | (0 0.5 1 1.5) |
| *R_6_’* | (3 4.6200 6.2400 7.8600) | (3 4.6200 6.2400 7.8600) | (3 4.6200 6.2400 7.8600) |
| *c_1_* | (132 32.6964 56.6148 1.5972) | (49.9416 24.3726 29.8086 17.3046) | (53.9580 31.3890 36.8250 24.3210) |
| *c_2_* | (31.2348 8.6658 14.1018 1.5978) | (0 23.4168 21.9252 15.7608) | (0 23.4168 21.9252 15.7608) |
